# Identifying Characteristics Associated with the Concentration and Persistence of Medical Expenses among Middle-Aged and Elderly Adults: Findings from the China Health and Retirement Longitudinal Survey

**DOI:** 10.3390/ijerph191912843

**Published:** 2022-10-07

**Authors:** Luyan Jiang, Qianqian Qiu, Lin Zhu, Zhonghua Wang

**Affiliations:** 1 School of Health Policy & Management, Nanjing Medical University, Nanjing 211166, China; 2 Public Health Policy and Management Innovation Research Group, Nanjing Medical University, Nanjing 211166, China; 3 Center for Global Health, Nanjing Medical University, Nanjing 211166, China

**Keywords:** medical expenditures, concentration, persistence, vulnerable groups, China

## Abstract

Medical expenses, especially among middle-aged and elderly people, have increased in China over recent decades. However, few studies have analyzed the concentration or persistence of medical expenses among Chinese residents or vulnerable groups with longitudinal survey data. Based on the data of CHARLS (China Health and Retirement Longitudinal Study), this study sought to identify characteristics associated with the concentration and persistence of medical expenses among Chinese middle-aged and elderly adults and to help alleviate medical spending and the operational risk of social medical insurance. Concentration was measured using the cumulative percentages of ranked annual medical expenses and descriptive statistics were used to define the characteristics of individuals with high medical expenses. The persistence of medical expenses and associated factors were estimated using transfer rate calculations and Heckman selection modeling. The results show that total medical expenses were concentrated among a few adults and the concentration increased over time. People in the high medical expense group were more likely to be older, live in urban areas, be less wealthy, have chronic diseases, and attend higher-ranking medical institutions. Lagged medical expenses had a persistent positive effect on current medical expenses and the effect of a one-period lag was strongest. Individuals with chronic diseases during the lagged period had a higher likelihood of experiencing persistent medical expenses. Policy efforts should focus on preventive management, more efficient care systems, improvement of serious illness insurance level, and strengthening the persistent protection effect of social medical insurance to reduce the high medical financial risk and long-term financial healthcare burden in China.

## 1. Background

The concentration and persistence of medical expenses are well documented [1,2,3]. Health care spending is overwhelmingly concentrated within a very small proportion of the population defined as high-cost users (HCUs) [4]. The persistence of medical expenditures generally refers to “long-term medical spending patterns” [3]. However, the concentration and persistence of medical costs have primarily been confirmed in the United States. Monheit et al. [5] found that 1% of patients account for 27% of annual medical spending in the US and Riley et al. [6] reported that 5% accounts for 34.4% of total health expenses. Kohn et al. [7] revealed that medical costs have significant persistence while Newhouse et al. [8] reported that 40% of current medical expenses are associated with past medical expenditures. Persistent high medical expenses have also been reported in many developed high-income countries, such as Germany, Canada, the Netherlands, and Australia [9,10,11,12]. Studies in developed Asian regions, such as Singapore and Japan, have reported that identifying the characteristics of high medical cost beneficiaries and long-term medical spending trends helps to reduce medical expenses when governments are faced with tighter medical budgets [13,14].

Studies have explored the persistence of medical expenses since the 1980s and have found that they can be anticipated by extended longitudinal profiles [8,15]. Other studies have shown that a relatively small number of spenders with persistent high medical costs account for about 20% of the medical expenses [2,5,12]. Researches have also focused on the impact of factors such as chronic diseases, demographic indicators, medical service utilization, and insurance, on the persistence and concentration of medical expenses [16,17]. Age, poor health status, and comorbidities are strong predictors of medical expense persistence [14,18], and researchers have highlighted the importance of early intervention to curb high costs [19,20].

With a rapidly ageing population and the changing spectrum of disease, medical expenses have risen significantly in recent years in both developed and developing countries [14,21]. As a small proportion of the population accounted for the majority of medical expenses and the persistence of medical expenditures could increase the operational risk of social medical insurance, previous literature empirically demonstrated that identifying the characteristics associated with the concentration and persistence of medical expenses was becoming a great concern around the world.

China is the largest developing country in the world. With improved medical technology and the universal coverage of social medical insurance in China, medical expenses have risen significantly in recent years. Since the new round of healthcare-system reform in 2009, China’s social medical insurance system has gradually improved with urban employee and urban and rural resident medical insurance now covering almost all citizens. The number of individuals with social medical insurance has reached 1.36 billion, the participation rate is more than 95%. The financing and reimbursement of social medical insurance have also improved, however, gaps in the level of medical security between urban and rural areas or other geographic regions remain. Thus, the wide coverage and increasing compensation of social medical insurance has resulted in higher medical expenses and increased healthcare utilization and cost inequalities. Peng et al. [21] indicated that nearly 50% of medical expenses in eastern China were paid by 10% of patients, and Ku L-JE et al. [22] demonstrated that being male, older, divorced, or having chronic diseases was associated with high medical spending. Xu et al. [23] also found that prior medical expenses had a positive persistent effect on current medical expenditures.

Despite many studies having examined the concentration and persistence of medical expenditures, there are still some gaps in this topic. First, most studies focus on general populations, while few have specifically assessed China or vulnerable groups. However, middle-aged and elderly people are more likely to suffer from chronic diseases and account for a larger share of total medical expenditures. Thus, it is particularly important to study the concentration and persistence of medical expenses among this at-risk group. To the best of our knowledge, no study has analyzed the concentration or persistence of medical expenses among middle-aged and elderly adults in China. Second, most studies on this topic in China have used the administrative data of social medical insurance. However, it is well known that the total medical expenses also include out-of-pocket medical expenditures and self-treatment expenses. In addition, some covariates, such as demographic variables, socioeconomic variables or medical service utilization indicators, cannot be obtained from these administrative data.

This study uses nationally representative elderly household survey data, which provides more comprehensive information of medical expenses (i.e., inpatient medical expenses, outpatient medical expenses, and self-treatment medical expenses), and more abundant covariates. The current study aims to identify the health and socioeconomic characteristics of this population with high medical spending and to establish dynamic regression models for medical expenses to evaluate the persistence and associated factors of medical costs. Findings could provide information about the concentration and persistence of medical expenses. In addition, this study provides the implication policies to reduce medical expenses and alleviate the operational risk of social medical insurance.

The remaining sections of this study are as follows. Section 2 introduces the approach of this study, including data source, data analysis, and definitions of the variables. Section 3 shows the descriptive results and the regression results. Section 4 provides the discussion and implication policy. Section 5 presents conclusions.

## 2. Methods

Based on previous studies on the concentration and persistence of medical expenses, the following hypotheses in this study are proposed:

**Hypothesis** **1:** *Medical expenses are concentrated in a small portion of middle-aged and elderly adults in China.*

**Hypothesis** **2:** *Significant persistence exists in the high medical expenses of middle-aged and elderly adults in China.*

**Hypothesis** **3:** *Chronic diseases and poor health status have a positive effect on the high medical expenses.*

**Hypothesis** **4:** *Chronic diseases and poor health status have a positive effect on the persistence of medical expenses.*

### 2.1. Data Source

Data were obtained from the China Health and Retirement Longitudinal Survey (CHARLS), a nationally representative survey of elderly household residents, conducted in 2013, 2015, and 2018 (https://charls.charlsdata.com/pages/Data/2013-charls-wave2/zh-cn, accessed on 18 November 2015; https://charls.charlsdata.com/pages/Data/2015-charls-wave4/zh-cn, accessed on 11 October 2017; https://charls.charlsdata.com/pages/Data/2018-charls-wave4/zh-cn, accessed on 14 September 2020). This survey, which covered 150 counties in 28 provinces, was used to construct a high-quality, nationally representative sample of Chinese household-dwelling adults aged ≥45 years for scientific research.

The survey used multi-stage stratified random sampling involving four steps. First, 150 county-level units covering 28 of the 30 provinces in mainland China, excluding Tibet, were sampled based on population size and stratified by GDP and district (urban or rural) using probability proportionate to size (PPS) sampling. Second, using the latest population data, village and community units within counties were chosen by referencing the National Bureau of Statistics. The administrative villages in rural areas and communities in urban areas were used as primary sampling units (PSUs). Three PSUs were selected within each county, and 450 PSUs were selected using the PPS. Third, approximately 25 household units were selected in each PSU based on sampling frames constructed using CHARLS GIS maps, considering the pre-investigation refusal rate. Fourth, one or two age-eligible respondents in each household were administered the survey and followed up every 2–3 years.

The CHARLS collects detailed information about each respondent and their spouse, including basic demographics, chronic disease status, health status and behaviors, health care utilization and insurance, income and retirement, and medical expenditures. Medical expenditure data include outpatient, hospitalization, and self-treatment expenses and total expenditures included the sum of all three spending categories. Based on CHARLS responses, nine chronic diseases were chosen based on their high prevalence or mortality rate in 2020 chronic disease status reports across China: hypertension, diabetes (i.e., high blood sugar), cancer or malignant tumors (except minor skin cancers), chronic lung disease (i.e., chronic bronchitis, emphysema), liver disease (except fatty liver, tumors or cancer), heart disease (i.e., coronary heart disease, angina, congestive heart failure, and other heart problems), kidney disease (except tumors or cancer), stomach and other digestive diseases (except tumors or cancer), and arthritis or rheumatism. Cross-sectional data from 2013, 2015, and 2018 were matched to a three-period dynamic panel dataset using ID, restricting the sample to individuals with data in each year. After dropping patients who had migrated or died, had negative expenditures, or had incomplete information, 19,869 observations (6623 samples) were included in the final dataset.

### 2.2. Data Analysis

#### 2.2.1. Determining the Concentration of Medical Expenses among Middle-Aged and Elderly Participants

Consistent with other surveys, most CHARLS survey respondents (47.2%) belonged to the “zero medical expenses” group. Participants with positive medical expenses were ranked from highest to lowest each year and divided into categories representing the top 1%, 10%, and 20%, and the bottom 50% of expenses. The medical expenses of each category were divided by the annual medical expenditures to estimate the cumulative percentage of each expense category and used to determine the concentration of total medical expenses as well as hospitalization, outpatient, and self-treatment-specific expenses from 2013 to 2018 [4,5,6,11].

#### 2.2.2. Determining the Persistence of High Medical Expenses among Middle-Aged and Elderly Participants

The persistence of high medical expenditures was determined by calculating the transfer rate of high medical expense samples [2,3,12,16]. Rates were estimated by determining the number of spenders in the top 10%, 20%, and 50% categories in 2013 that transferred into other categories or remained in the original categories in 2015 and 2018 and then dividing these values by the number of spenders in the top 10%, 20%, and 50% categories in 2013. The top 1% category was excluded because its small sample size made it difficult to obtain scientifically representative transfer rates.

#### 2.2.3. Descriptive Analysis

Descriptive statistics were used to analyze the characteristics of middle-aged and elderly people with high medical costs. While the top 1%, 10%, and 50% categories were used to estimate the concentration of medical expenses, individuals in the top 10% and 20% of annual total medical expenses were used for the descriptive analysis because the top 1% group was too small to calculate reliable rates for specific characteristics. Participants with the top 10% of total medical costs in 2013, 2015, and 2018 were combined as the “Top 10% high medical expense group” while those with the bottom 90% of total medical expenses in 2013, 2015, to 2018 were combined as the “Bottom 90% medical expense group” and defined as the control group. Similarly, a “Top 20% high medical expense group” and “Bottom 80% medical expense group” were also created using combined data from each of the survey years.

#### 2.2.4. Regression Analysis of Medical Expenses among Middle-Aged and Elderly Participants

As a result of the large proportion of participants in the “zero medical expenses” group and the “long-tail distribution” of positive medical costs, the Heckman selection model was used to identify the contribution of related variables to medical spending incidence. Lagged medical expenditures were used as the independent variable to analyze the persistence of medical expenses. The analysis included a regression of medical expense incidence and a regression of total medical expenditures.

The first regression used the probit model for estimation as follows:

(1) $$I_{it}=\alpha +\left(X_{it}\beta \right)+\sum_{k}\gamma_{t-k}I_{i,t-k}+\sum_{k}\sum_{j}\lambda_{j,t-k}I_{i,t-k}D_{ij,t-k}+\upsilon_{it}$$

In this model, the dependent variable, “incidence of medical costs” ($I_{it}$), is a binary variable defined as “whether an individual *i* had medical expenses in year t” and dichotomized as “0” = “Non-incurrence of medical expenses” or “1” = “Incurrence of medical expenses.” $I_{i,t-k}$ denotes the lag term of “whether medical expenses occurred.” $X_{it}$ are a series of control variables representing the demographic factors, age, sex, education level, marital status, retirement condition, and residence. $D_{ij,t-k}$ is a dummy indicator representing “whether individual *i* suffered from disease *j* in lagged *k* period.” $I_{i,t-k}D_{ij,t-k}$ are cross-variables indicating “whether medical expenses incurred in the lag period” interacted with “the prevalence of diseases *i* in the lag k stage.” In the current paper, *k* = 1,2, or *k* here does not refer to the specific value of the year. Instead, *t*-1 denotes lagged one period while *t*-2 denotes lagged two periods. $\upsilon_{it}$ is the error term, with a normal distribution, and γ and λ are the regression coefficients assessed in this study. While γ assesses the persistence of incurring medical expenses, λ represents the effect of diseases in the lagged period on the incidence of current medical expenses. Based on the study of Bai et al., we reported “*dy/dx*” as the marginal effect coefficients of the probit regression on incidence of medical expenses [24].

The second model represented the total medical expenditures and OLS regression was used, as follows:

(2) $$\ln(Y_{it}|I_{it}>0)=\delta +\left(Z_{it}\varphi \right)+\sum_{k}\eta_{t-k}\ln(1+Y_{i,t-k})+\varepsilon_{it}$$

In this model, the dependent variable,$Y_{it}$, is the total medical expenditure of an individual *i* in year *t*, and was made logarithmic to normalize the skewed medical expense data. The key independent variables, $Y_{i,t-k}$, are “the lagged term of medical expenses for each individual *i* over a certain number of years.” For example, if $Y_{i,t-k}$ includes $Y_{i,2015}$ and $Y_{i,2013}$ for 2018 then *k* = 2. According to Peng et al. [21], $\ln\left(1+Y_{i,t-k}\right)$ is the logarithm of $Y_{i,t-k}$. If $Y_{i,t-k}$ = 0, 1 should be added when transforming into a logarithm, and if $Y_{i,t-k}$ is not equal to 0, the log of the original lagged expenditure, k, should be used to define Equation (1). $Z_{it}$ is a vector for covariates such as socioeconomic and other endogenous factors, φ and $\eta_{t-k}$ are the regression coefficients of the equation, $\eta_{t-k}$ determines how many percent the value of $Y_{it}$ will change if $Y_{i,t-k}$ changes by 1%, and $\varepsilon_{it}$ is a random error term. Statistical analyses were performed using Stata, version 16.0 (Stata Corp, Inc., Cary, TX, USA).

#### 2.2.5. Variable Selection

##### Dependent Variables

The dependent variables included total medical expenditures (*Y*) and annual incidence of medical costs (*I*). Annual total medical costs were calculated by combining the outpatient, hospitalization (fees paid to the hospital, including ward fees but not wages paid to a hired nurse, transportation costs, and accommodation costs for oneself or family members), and self-treatment expenses (medicines purchased by the patient but not including prescription medications). *I* is a dummy binary variable set based on *Y* such that if the total medical expenses are not equal to zero, *I* is defined as “1”, otherwise it is defined as “0.”

##### Independent Variables

The independent indicators from three dimensions (predisposing factors, enabling factors, and need factors) were selected using the Anderson health service utilization model [25], the mainstream model used for health service utilization research. The model divides factors affecting health service utilization into three categories: predisposing, enabling, and need factors. Predisposing factors include demographic and social structure factors, such as age, sex, marital status, retirement condition, education level, and residence. Enabling factors include financial and organizational variables such as household income per capita, health insurance, inpatient visit times, type of outpatient institution and visit times, and multi-type outpatient facility visits. Specifically, “multi-type outpatient facility visits” is a virtual variable based on the multi-choice question “Which types of medical facilities have you visited in the last 4 weeks for outpatient treatment?” The outpatient institutions were divided into two categories based on the grade classification of the medical facilities. While general, specialized and Chinese medicine hospitals = 0, community health centers, town hospitals, and village clinics = 1. If the respondent received treatment from both high-ranking hospitals and primary medical institutions, “type of outpatient institution” = 1, and if the respondent only received medical services from primary medical institutions, the “type of outpatient institution” = 0. Need factors included self-reported health status and chronic diseases. To investigate the impact of several chronic diseases, a “comorbidity” dummy variable was developed. All independent variables were set as categorical indicators. Detailed descriptions of the samples are included in Table 1.

## 3. Results

### 3.1. The Concentration of Medical Expenses among Middle-Aged and Elderly Participants

**Figure 1:**
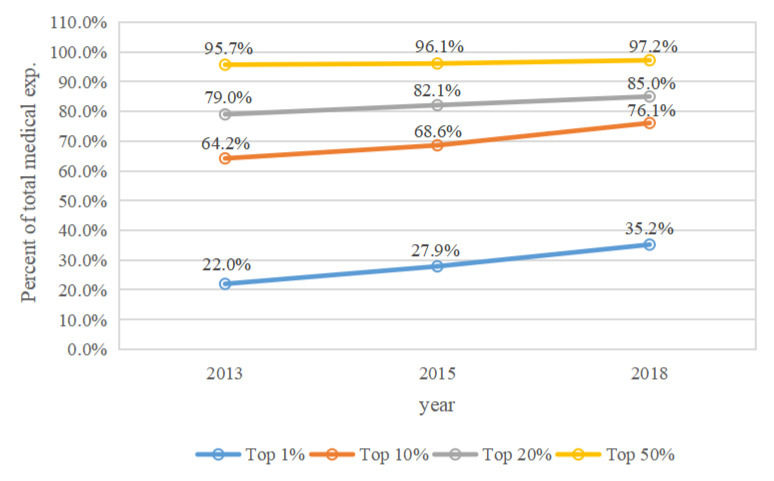
The concentration of total medical expenses by year.

**Figure 2:**
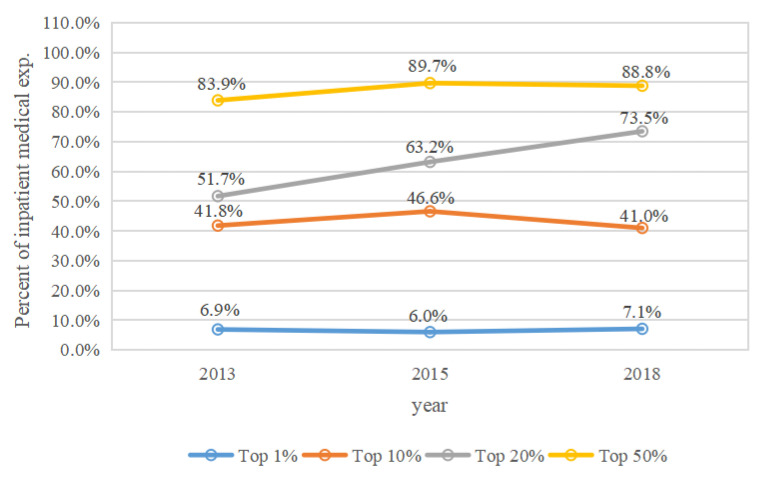
The concentration of inpatient medical expenses by year.

**Figure 3:**
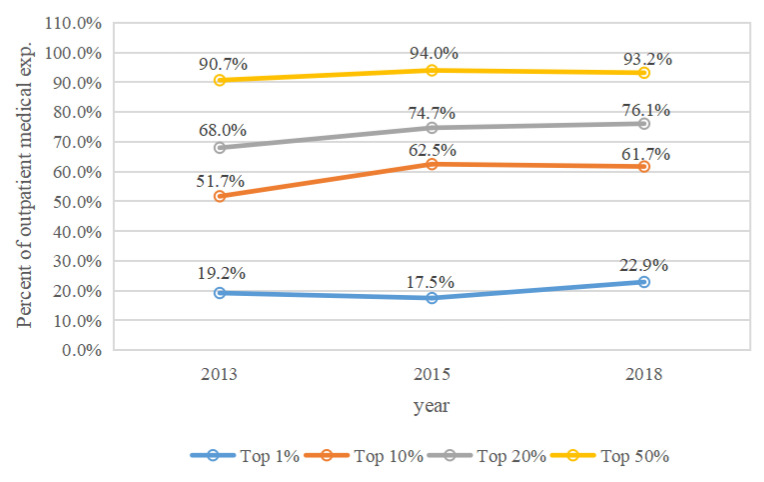
The concentration of outpatient medical expenses by year.

**Figure 4:**
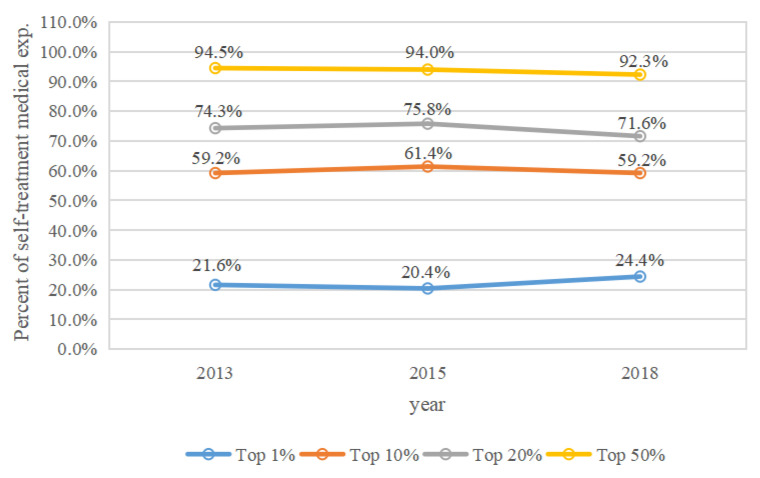
The concentration of self-treatment medical expenses by year.

The cumulative percentages of medical expenditures spent by middle-aged and elderly people who were in the top 1%, 10%, 20%, and 50% annual medical expense categories in 2013, 2015, and 2018 are shown in Figure 1, Figure 2, Figure 3 and Figure 4. Four types of expenses were reported: total medical expenses, and inpatient, outpatient, and self-treatment medical expenses.

In 2013, 2015, and 2018, middle-aged and elderly people in the top 1% category of total medical expenditures spent 22.0%, 27.9%, and 35.2% of the total annual medical expenses, respectively (Figure 1). Medical expenses for middle-aged and elderly people in the top 10% category accounted for 64.2%, 68.6%, and 76.1% of the total medical costs, respectively. Those with the top 20% of expenditures spent 79.0%, 82.1%, and 85.0% of the total annual medical expenses, respectively, while the share of spending on the total medical expenditures of those with expenses in the top 50% was above 95% in 2013, 2015, and 2018. The proportion of total medical expenses spent by the top 1%, 10%, 20%, and 50% of middle-aged and elderly spenders increased over time.

During 2013, 2015, and 2018, middle-aged and elderly people with the top 1% of hospitalization expenses spent 6.9%, 6.0%, and 7.1% of annual hospitalization costs, respectively (Figure 2). Those with the top 10% of expenditures spent 41.8%, 46.6%, and 41.0% of the annual inpatient expenses in 2013, 2015, and 2018, respectively. Medical expenses for middle-aged and elderly people in the top 20% category accounted for 51.7%, 63.2%, and 73.5% of the annual inpatient expenditures, respectively, while those with the top 50% spent 83.9%, 89.7%, and 88.8%, respectively. The proportion of individuals with the top 1%, 10%, 20%, and 50% of expenditures fluctuated from 2013 to 2018.

During 2013, 2015, and 2018, middle-aged and elderly people with the top 1% of outpatient expenses spent 19.2%, 17.5%, and 22.9% of the annual costs, respectively (Figure 3). Those with the top 10% spent 51.7%, 62.5%, and 61.7% of the annual outpatient expenses in 2013, 2015, and 2018, respectively. Those with the top 20% spent 68.0%, 74.7%, and 76.1%, respectively, while those with the top 50% spent 90.7%, 94.0%, and 93.2%, respectively. The proportion of individuals with the top 1%, 10%, 20%, and 50% outpatient expenses demonstrated an increasing trend from 2013 to 2018.

In 2013, 2015, and 2018, middle-aged and elderly people with the top 1% of self-treatment expenses spent 21.6%, 20.4%, and 24.4% of the annual costs, respectively (Figure 4). Those with the top 10% spent 59.2%, 61.4%, and 59.2% of the annual self-treatment medical costs in 2013, 2015, and 2018, respectively. Those with the top 20% spent 74.3%, 75.8%, and 71.6%, respectively, and those with the top 50% spent 94.5%, 94.0%, and 92.3%, respectively. The proportion of individuals with the top 1%, 10%, 20%, and 50% annual self-treatment medical expenses fluctuated slightly from 2013 to 2018.

### 3.2. The Characteristics of Middle-Aged and Elderly Participants with High Medical Expenses

There were significant differences between the characteristics of the middle-aged and elderly participants with the top 10% and 20% medical expenditures and those with the bottom 80% and 90% medical expenditures (Table 2 and Table 3). Those in the top 10% medical expense group were more likely to be female (56.84% vs. 50.86%; *p* < 0.000), older (age ≥75 years: 9.56% vs. 7.35%; *p* < 0.000), retired (17.34% vs. 9.66%; *p* < 0.000), more educated (tertiary education: 1.07% vs. 0.44%; *p* < 0.000), and to live in an urban area (28.78% vs. 22.50%; *p* < 0.000) than those in the bottom 90% medical expense group. Middle-aged and elderly adults with the top 10% medical expenses were more likely to have a lower income level (household income per capita ≤CNY 8000: 83.20% vs. 76.41%; *p* < 0.000), receive outpatient treatment in higher-ranking hospitals (58.18% vs. 19.71%; *p* < 0.000), or have multi-types of outpatient visits (8.67% vs. 0.51%; *p* < 0.000). In addition, middle-aged and elderly participants with the top 10% medical expenses tended to have more hospitalizations (number of hospitalizations >1: 28.87% vs. 0.90%; *p* < 0.000), outpatient treatments (number of outpatient visits ≥36: 17.78% vs. 1.17%; *p* < 0.000), and multiple chronic diseases (comorbidity: 65.95% vs. 36.71%).

The top 10% of medical spenders demonstrated a significantly higher prevalence of chronic diseases (hypertension: 36.73% vs. 24.52%, diabetes: 12.24% vs. 5.65%, cancer: 2.86% vs. 0.97%, chronic lung diseases: 19.48% vs. 9.00%, liver diseases: 10.01% vs. 4.03%, heart diseases: 29.31% vs. 11.35%, kidney diseases: 12.96% vs. 5.67%, stomach or digestive diseases: 36.37% vs. 22.29%, arthritis or rheumatism: 48.70% vs. 33.77%) than the bottom 90% of medical spenders (Table 3).

### 3.3. Persistence of High Medical Expenses

No middle-aged and elderly people with the top 10% medical expenses in 2013 remained in the top 10% in 2015 or 2018 (Table 4). Similarly, only 30.83% and 26.46% of those with the top 20% medical expenses in 2013 still had the top 20% expenses in 2015 and 2018, respectively. In contrast, 46.42% and 50% of those with the top 50% medical expenses in 2013 still had the top 50% expenses in 2015 and 2018.

Most (70%) of those with the top 10% medical expenses in 2013 transferred to the top 20% in 2015 and 2018, and 80% and 70% of those with the top 10% high medical expenses transferred to the top 50% in 2015 and 2018, respectively. In contrast, 20% and 30% of participants with the top 10% of medical expenses in 2013 transferred to the bottom 50% in 2015 and 2018, and 55.03% and 55.29% of those with the top 20% of medical expenses in 2013 transferred to the top 50% of medical expenses in 2015 and 2018. While 44.97% and 44.71% of those with the top 20% of medical expenses in 2013 transferred to the bottom 50% in 2015 and 2018, respectively, 21.48% and 20.64% with the top 50% medical expenses in 2013 transferred to the top 20% in 2015 and 2018, respectively, and 53.58% and 50% of those with the top 50% medical expenses in 2013 transferred to the bottom 50% in 2015 and 2018.

### 3.4. Heckman Selection Model Regression

#### 3.4.1. Incidence of Medical Expenses

Results of the two regression models, including the lagged one-period and two-period variables, are shown in Table 5. Once medical expenses occurred, the probability of incurring persistent medical expenditures in the next period increased significantly by 16.5% and 15.8%, respectively. Incurring medical expenses in lagged two periods increased the incidence of current medical expenses by 11.7%. Females had a significantly higher likelihood of incurring medical expenses than males (dy/dx = 0.033, 0.027), and chronic diseases had a persistent effect on the incidence of medical expenses. The probability of incurring medical expenses in the current period increased significantly by 29.3% once middle-aged and elderly adults with cancer had medical expenses in lagged one period (dy/dx = 0.293). Meanwhile, those suffering from hypertension (dy/dx = 0.102, 0.069), diabetes (dy/dx = 0.098), chronic lung diseases (dy/dx = 0.078, 0.081), liver diseases (dy/dx = 0.049), heart diseases (dy/dx = 0.074), kidney diseases (dy/dx = 0.089, 0.115), digestive diseases (dy/dx = 0.055) and arthritis or rheumatism (dy/dx = 0.060) in the lagged one-period significantly increased the probability of incurring medical expenses. In addition, suffering from hypertension, liver disease, arthritis, or rheumatism in the lagged two-period also increased the incidence of current medical costs (dy/dx = 0.103, 0.111, and 0.036, respectively).

#### 3.4.2. Total Medical Expenses

Factors associated with total medical expenses among middle-aged and elderly participants in the second part of Heckman selection model are shown in Table 6. The medical expenses were logarithmically transformed in the regression model. When other variables were controlled, the current level of medical expenses was strongly affected by past medical expenditures. For each 10% increase in medical costs during the lagged one-period, the current medical expenses increased by 2.25% and 1.65%, respectively, and for each 10% increase in medical expenses during the lagged two-period, current medical expenses increased by 1.13%.

Middle-aged and elderly people who were ≥75 years of age generally had more medical expenses than those <55 years of age (Coef. = 0.447). Females had higher medical expenses than males (Coef. = 0.328, 0.297) and married respondents had higher medical expenses than the unmarried (Coef. = 0.258). Respondents living in urban areas generally spent more on medical expenses than those living in rural areas (Coef. = 0.221).

Middle-aged and elderly adults with more outpatient visits had higher medical costs (Coef. = 24–36 and ≥36 outpatient visits = 0.331, 0.827) as did those with more hospitalizations (Coef. = 1.067, 0.911). In addition, respondents receiving medical services in primary medical institutions had significantly lower medical costs than those seen in general or specialized hospitals (Coef. = −1.167, −1.151). Medical expenses were significantly higher for middle-aged and elderly individuals with a household per capita income of CNY15,600–30,000 and ≥CNY 30,000 than those with an income ≤CNY 8000 (Coef. of CNY 15,600–30,000 and ≥CNY 30,000 = 0.284, 0.307). In addition, respondents with health insurance generally spent more on medical expenses (Coef. = 2.734, 2.012). A significantly positive association was found between those with chronic diseases and higher medical expenses. Respondents with hypertension (Coef. = 0.383, 0.315), diabetes (Coef. = 0.408, 0.328), chronic lung diseases (Coef. = 0.220), heart disease (Coef. = 0.346, 0.308), and arthritis or rheumatism (Coef. = 0.197) generally had higher medical expenses. The total medical expenses for those with comorbidities were significantly higher (Coef. = 0.274, 0.654). In addition, middle-aged and elderly adults with poor self-reported health status had higher medical costs (Coef. = 1.388, 1.811).

## 4. Discussion

The medical expenses of middle-aged and elderly people account for a large proportion of all medical expenses because of the higher incidence of age-related chronic disease in this population. Thus, it is important to study the concentration and persistence of medical expenses in this group. To the best of our knowledge, this study of a nationally representative longitudinal Chinese household survey population is the first to analyze the extent and characteristics of the concentration and persistence of medical expenses among Chinese middle-aged and elderly adults [21,22,23].

### 4.1. The Concentration of Medical Expenses among Middle-Aged and Elderly Participants

This study found that 22.0–35.2% of total annual medical expenses were spent by the top 1% of middle-aged and elderly medical spenders, while 64.2–76.1% were spent by the top 10%, 79.0–85.0% were spent by the top 20%, and 95.7–97.2% were spent by the top 50%. These findings demonstrate that total medical expenses are concentrated in a small portion of middle-aged and elderly adults, supporting findings from previous studies [6,10]. This means that hypothesis 1 is proven. These results indicate that there is an inequitable utilization of health resources among middle-aged and elderly adults. As a result, policy efforts should focus on optimizing the allocation of health service utilization by different groups to enhance social welfare [26]. This study suggest: (1) To achieve the similar financing and reimbursement level of social medical insurance between urban and rural regions, the Urban Employee Basic Medical Insurance and the New Rural Cooperation Medical Insurance should be further integrated. (2) To narrow the gap in allocation of medical resources among regions, the government should improve the financial transfer payment system. (3) The government should improve the security level of serious illness insurance and medical assistance among rural residents and the low-income population. (4) To integrate medical resources and reduce the excessive utilization of medical services, medical consortiums need to be further established, and standardization of the clinical care process should be continuously implemented.

The concentration of total medical expenses increased from 2013 to 2018, which contrasts with some studies [4,6]. This suggests that the total medical expenses of Chinese middle-aged and elderly people were more concentrated among a small number of individuals in recent years, which may be the result of advancements in medical treatment technologies and universal social health insurance coverage in China. Thus, differentiated measures of social health insurance for high medical expense spenders among these demographics should be a priority concern for public policy. Meanwhile, the concentration of outpatient medical expenses also increased from 2013 to 2018. Inpatient medical expenses in the top 10% decreased slightly from 2015 to 2018, this may be the result of recent reforms to social medical insurance and standardization of the clinical care process in China. The concentration of self-treatment medical expenses for participants in the top 1% increased from 2015 to 2018, likely as a result of worsening inequities in self-treatment expenses. We suggest that the fairness of self-treatment can be better improved in the following ways. To reduce the excessive utilization of over-the-counter medications, general practitioners can guide the use of these medicines. Health administrative departments, price bureaus, and drug administrations should reasonably adjust the prices of self-medication drugs, health supplements, and health care equipment.

### 4.2. Persistence of High Medical Expenses among Middle-Aged and Elderly Participants

Some middle-aged and elderly participants with high medical expenses in 2013 remained in the same high medical expense categories in 2015 and 2018, supporting prior studies illustrating the persistence of high medical costs [7,22,27]. Hypothesis 2 is supported. This may be linked to the transitioning disease spectrum and high prevalence of chronic diseases among high medical expense groups. Policy efforts should focus on preventive management for individuals at risk of incurring high medical expenses [2,6,20].

Most middle-aged and elderly participants with the top 10% of medical expenses in 2013 transferred to the top 20% or 50% categories in 2015 and 2018. This is consistent with previous surveys [17,28] and indicates that high medical expenses may have been brought under control with reform to the medical system and the advancement of medical technology in China [12,15]. However, it should be noted that almost 20% of middle-aged and elderly adults in the top 50% spending category in 2013 transferred to the top 20% spending category in 2015 and 2018. Thus, policy efforts will need to better define the characteristics of this group to effectively control rising medical expenses.

### 4.3. Characteristics of Middle-Aged and Elderly Participants with High Medical Expenses

Middle-aged and elderly participants with high medical expenses were more likely to be female and older, which is in agreement with previous studies [5,29]. This may be the result of the increasing incidence of menopause and severe disease in older women [30,31]. A high education level was also associated with higher medical expenses, possibly resulting from better health awareness and a stronger motivation to seek medical services [32]. In addition, participants with high medical expenses were more likely to live in urban than rural areas. This may be attributed to socioeconomic differences between urban and rural areas in China [33]. Urban residents tend to have higher incomes and are thus able to incur higher medical expenses and advanced medical technologies and resources tend to be more concentrated in urban areas. Thus, initiatives that optimize the allocation of health resources in urban and rural areas should be considered to narrow the gap between these regions. Participants with lower income were also more likely to be in the higher medical expenses group, which is consistent with some studies [11,34]. This may be because less-wealthy groups have lower health management awareness and more risky lifestyle behaviors that increase the prevalence of severe diseases [35]. Thus, health sectors should strengthen health management, disease prevention, and financial assistance for poorer individuals.

In this study, participants receiving medical services in general or specialized hospitals had high medical expenses, potentially as a result of excessive medical treatment. This should be addressed by reforming hierarchical health services and promoting rational utilization. Middle-aged and elderly participants who visited multi-type medical institutions also demonstrated high medical expenses. Social health insurance policies should focus on reducing the medical expenses of these at-risk groups.

Middle-aged and elderly people with hypertension, diabetes, cancer, chronic lung diseases, liver diseases, heart diseases, kidney diseases, digestive diseases, arthritis, rheumatism, and comorbidities were more likely to have high medical expenses, which supports previous survey findings [17,36]. Hypothesis 3 is proven. Prevention management and early screening and treatment for chronic diseases should help to reduce medical expenses in this population.

### 4.4. Factors Associated with the Persistence of Total Medical Expenses

This study found that the incidence of medical expenses for middle-aged and elderly people increased by 15.8–16.5% from 2013 to 2015 and by 11.7% from 2013 to 2018. Current medical expenses increased by 1.65–2.25% and 1.13% for every 10% increase in the lagged one and lagged two-period medical expenses, respectively, which is consistent with previous survey results [5,37]. These findings demonstrate that medical expenses are significantly persistent. While previous medical expenses had a persistently positive effect on current medical expenses, the effect of the lagged one period was the strongest, which is also supported by other studies [15,18]. This effect may be attributed to the long-term treatment of chronic diseases. It is worth noting that middle-aged and elderly people with diabetes, cancer, chronic lung diseases, heart diseases, kidney diseases, and digestive diseases had a high likelihood of having persistent medical expenses for two periods, while those with hypertension, liver diseases, arthritis, or rheumatism were more likely to have persistent medical expenses for three periods. In addition, those with hypertension, diabetes, chronic lung diseases, heart diseases, arthritis, rheumatism, or comorbidities generally had higher medical expenses, supporting prior studies [16,38]. Hypothesis 4 is proven. To control persistent medical expenses and higher medical expenses, preventive measures should focus on reducing the incidence of chronic diseases. Health sectors should consider joining primary medical institutions to establish persistent integrated care systems for middle-aged and elderly people with chronic diseases to reduce the overuse of medical resources during long-term treatment regimens. In addition, the reimbursement of medical insurance should be improved for those with chronic diseases to alleviate persistent medical expense burdens.

This study found that females, with age ≥75 years, and living in an urban area were all positive factors for higher medical expenses, which is consistent with other survey results [21,39]. In addition, married individuals had higher medical costs than unmarried, possibly because married people utilize more health services as a result of supervision from their spouses. Middle-aged and elderly people with high per capita income and health insurance generally incurred higher medical expenses, indicating that those with high income could afford higher medical expenses. Moreover, social health insurance may promote an increase in medical expenses to some extent. Participants who received more outpatient visits and hospitalizations or who visited higher-ranking hospitals generally had higher medical expenses, which is consistent with a previous study [40]. Thus, increasing the reimbursement level of social medical insurance of hierarchical health service in China may help to reduce the financial healthcare burden of high-cost users.

There are some limitations to the current study. Because the CHARLS does not include information on chronic disease diagnoses, the chronic disease prevalence was obtained by asking adults whether they had been diagnosed with a chronic disease. Thus, individuals who might have had a chronic disease but were not or did not recall being diagnosed may have been excluded in which case the true prevalence of a particular condition may be underestimated. In addition, data on missing samples were removed from the study, potentially resulting in an undercount of individuals with persistent medical expenses. Finally, medical expenses are likely to be influenced by additional factors which are not included in the claims data. Future research could consider further exploring the influence mechanism of the persistence of high medical expenses in more complex models.

## 5. Conclusions

This study found that total medical expenses were concentrated among a few middle-aged and elderly individuals and the concentration increased over time. Some health and socioeconomic characteristics, such as chronic disease, age, education level, residence, employment status, income level, and medical service utilization condition, were significantly associated with high medical expenses. Thus, the government should address these factors to further improve security levels of serious illness insurance among middle-aged and elderly participants with high medical expenses to lower medical financial risk. High medical expenses also demonstrated strong persistence. Lagged medical expenses had a significantly persistent positive effect on current medical expenses and the effect of lagged one period was the strongest. Those having chronic diseases in the lagged periods were more likely to have persistent medical expenses. To address this, the government should establish a more efficient care system and strengthen the persistent protection effect of social health insurance policies to alleviate long-term financial healthcare burdens.

## Figures and Tables

**Table 1:** Description of the dependent and independent variables.

Variable	Category	Indicator/Survey Question
Dependent variables		
Total medical expenses	CNY	Question: What was the total medical expenses during the past year?
Incidence of medical expenses	=0, non-incurrence of medical expenses; =1, incurrence of medical expenses	Whether the total medical expenses were equal to zero.
Independent variables		
Predisposing factors		
Sex	=0, male; =1, female	
Age (years)	=0.45–55; =1.55–65; =2.65–75; =3, if ≥ 75	
Marital status	=0, not married; =1, married	Question: What is your marital status? (Separation, divorce, widowhood, and cohabitation belong to “not married”.)
Retirement status	=0, not retired; =1, retired	Question: What is your retirement status?
Education level	=0, less than lower secondary; =1, upper secondary, vocational training; =2, tertiary	Question: What is the highest level of education you have attained?
Area of residence	=0, rural; =1, urban	Type of residence.
Enabling factors		
Income level	=0, ≤CNY 8000; =1, CNY 8,000–15,600; =2, CNY 15,600–30,000; =3, ≥CNY 30,000	Yearly household income divided by the number of household members.
Social health insurance	=0, have no insurance; =1, have insurance	Question: Do you have any social health insurance?
Type of outpatient medical facilities	=0, general hospital, specialized hospital, Chinese medicine hospital; =1, community healthcare center, township hospital, village clinic	Question: Which types of medical facilities have you visited in the last 4 weeks for outpatient treatment?
Multi-type outpatient facility visits	=0, no multi-type out- patient facility visits; =1, muti-type outpatient facility visits	Whether attended multiple types of outpatient facilities.
Number of outpatient visits	=0, ≤24; =1, 24–36; =2, ≥36	Question: How many times did you visit/been visited by during the last month?
Number of hospitalizations	=0, ≤1; =1, >1	Question: How many times have you received inpatient care during the past year?
Need factors		
Self-reported health status	=0, very good; =1, good; =2, fair; =3, poor	
Comorbidity	=1, ≤1; =2, >1	Number of chronic diseases.
Hypertension	=0, no; =1, yes	
Diabetes	=0, no; =1, yes	
Cancer	=0, no; =1, yes	Excluding minor skin cancers
Chronic lung diseases	=0, no; =1, yes	Excluding tumors or cancer
Liver diseases	=0, no; =1, yes	Excluding fatty liver, tumors, cancer
Heart diseases	=0, no; =1, yes	
Kidney diseases	=0, no; =1, yes	Excluding tumors or cancer
Stomach or other digestive diseases	=0, no; =1, yes	Excluding tumors or cancer
Arthritis or rheumatism	=0, no; =1, yes	

**Table 2:** Characteristics of middle-aged and elderly participants with the top 10% and 20% of medical expenses.

	Top 10% *n* = 1119 (5.63%)	Bottom 90% *n* = 18,750 (94.37%)	*p*	Top 20% *n* = 2180 (10.97%)	Bottom 80% *n* = 17,689 (89.03%)	*p*
Predisposing factors						
Sex			0.000			0.000
Male	43.16%	49.14%		42.89%	49.53%	
Female	56.84%	50.86%		57.11%	50.47%	
Age (years)			0.000			0.000
45–55	23.77%	28.79%		25.28%	28.91%	
55–65	38.25%	40.12%		38.53%	40.20%	
65–75	28.42%	23.73%		26.51%	23.69%	
≥75	9.56%	7.35%		9.68%	7.20%	
Marital status			0.817			0.374
Not married	11.80%	11.57%		12.16%	11.51%	
Married	88.20%	88.43%		87.84%	88.49%	
Retirement status			0.000			0.000
Not retired	82.66%	90.34%		83.62%	90.68%	
Retired	17.34%	9.66%		16.38%	9.32%	
Education level			0.000			0.002
Less than lower secondary	85.08%	88.82%		86.79%	88.83%	
Upper secondary, vocational training	13.85%	10.74%		12.39%	10.74%	
Tertiary	1.07%	0.44%		0.82%	0.43%	
Area of residence			0.000			0.000
Rural	71.22%	77.50%		71.74%	77.81%	
Urban	28.78%	22.50%		28.26%	22.19%	
Enabling factors						
Income level			0.000			0.000
≤CNY 8000	83.20%	76.41%		81.19%	76.25%	
CNY 8000–15,600	4.29%	7.66%		5.55%	7.71%	
CNY 15,600–30,000	5.36%	7.76%		5.55%	7.88%	
≥CNY 30,000	7.15%	8.18%		7.71%	8.17%	
Social health insurance			0.456			0.455
Have no insurance	1.97%	2.31%		2.06%	2.32%	
Have insurance	98.03%	97.69%		97.94%	97.68%	
Type of outpatient medical facilities			0.000			0.000
General hospital, specialized hospital, Chinese medicine hospital	58.18%	19.71%		48.46%	13.87%	
Community healthcare center, township hospital, village clinic	41.82%	80.29%		51.54%	86.13%	
Multi-type outpatient facility visits			0.000			0.000
No multi-type outpatient facility visits	91.33%	99.49%		93.39%	99.72%	
Multi-type outpatient facility visits	8.67%	0.51%		6.61%	0.28%	
Number of outpatient visits			0.000			0.000
≤24	72.39%	97.60%		77.20%	98.52%	
24–36	9.83%	1.23%		8.76%	0.84%	
≥36	17.78%	1.17%		14.04%	0.64%	
Number of hospitalizations			0.000			0.000
≤1	71.13%	99.10%		80.73%	99.60%	
>1	28.87%	0.90%		19.27%	0.40%	
Need factors						
Self-reported health status			0.000			0.000
Very good	3.84%	14.31%		3.72%	14.96%	
Good	6.70%	15.17%		7.11%	15.63%	
Fair	39.59%	53.00%		44.54%	53.20%	
Poor	49.87%	17.51%		44.63%	16.21%	
Comorbidity			0.000			0.000
Without comorbidity	34.04%	63.29%		36.19%	64.78%	
With comorbidity	65.95%	36.71%		63.81%	35.22%	

Note: Consolidated observations in the top 10% and top 20% medical expense groups from 2013 to 2018 were defined as the “Top 10%” and “Top 20%” medical spending groups, respectively. “*p*” indicates the significance of the difference test between subgroups.

**Table 3:** Chronic diseases in middle-aged and elderly participants with the top 10% and 20% of medical expenses.

	Top 10% *n* = 1119 (5.63%)	Bottom 90% *n* = 18,750 (94.37%)	*p*	Top 20% *n* = 2180 (10.97%)	Bottom 80% *n* = 17,689 (89.03%)	*p*
Hypertension			0.000			0.000
No	63.27%	75.48%		63.35%	76.21%	
Yes	36.73%	24.52%		36.65%	23.79%	
Diabetes			0.000			0.000
No	87.76%	94.35%		88.67%	94.63%	
Yes	12.24%	5.65%		11.33%	5.37%	
Cancer			0.000			0.000
No	97.14%	99.03%		97.71%	99.08%	
Yes	2.86%	0.97%		2.29%	0.92%	
Chronic lung diseases			0.000			0.000
No	80.52%	91.00%		82.25%	91.41%	
Yes	19.48%	9.00%		17.75%	8.59%	
Liver diseases			0.000			0.000
No	89.99%	95.97%		91.10%	96.20%	
Yes	10.01%	4.03%		8.90%	3.80%	
Heart diseases			0.000			0.000
No	70.69%	88.65%		74.17%	90.43%	
Yes	29.31%	11.35%		25.83%	9.57%	
Kidney diseases			0.000			0.000
No	87.04%	94.33%		87.57%	94.70%	
Yes	12.96%	5.67%		12.43%	5.30%	
Stomach or other digestive diseases			0.000			0.000
No	63.63%	77.71%		64.77%	78.41%	
Yes	36.37%	22.29%		35.23%	21.59%	
Arthritis or rheumatism			0.000			0.000
No	51.30%	66.23%		50.37%	67.25%	
Yes	48.70%	33.77%		49.63%	32.75%	

Note: Consolidated observations in the top 10% and top 20% medical expense groups from 2013 to 2018 were defined as the “Top 10%” and “Top 20%” medical spending groups, respectively. “*p*” indicates the significance of the difference test between subgroups.

**Table 4:** Persistence of total medical expenses during 2013, 2015, and 2018.

Expense Ranking in 2013	Expense Ranking in 2015			Expense Ranking in 2018		
	Top 10%	Top 20%	Top 50%	Top 10%	Top 20%	Top 50%
Top 10%	0	70.00%	80.00%	0	70.00%	70.00%
Top 20%	0.26%	30.82%	55.03%	0.40%	26.46%	55.29%
Top 50%	0.28%	21.48%	46.42%	0.22%	20.64%	50.00%

**Table 5:** Heckman probit coefficients: Regression on incidence of medical expenses.

	(1)		(2)	
	dy/dx	Std. Err.	dy/dx	Std. Err.
Lagging item of with or without expenses (ref. without)				
L1	0.165 ***	0.010	0.158 ***	0.015
L2			0.117 ***	0.015
Demographic variables				
Age (ref. 45–55)				
55–65	−0.009	0.011	−0.013	0.016
65–75	0.007	0.012	0.011	0.017
≥75	0.019	0.017	0.015	0.023
Gender (ref. male)	0.033 ***	0.008	0.027 **	0.012
Education (ref. less than lower secondary)				
Upper secondary, vocational training	−0.012	0.014	−0.022	0.020
Tertiary	−0.030	0.063	−0.016	0.096
Marriage (ref. not)	−0.001	0.013	−0.003	0.018
Employ (ref. not)	0.006	0.014	−0.021	0.019
Residence (ref. rural)	0.008	0.012	−0.017	0.018
Lagging item of with or without expenses*disease (ref. without)				
L1*Hypertension	0.102 ***	0.014	0.069 ***	0.025
L1*Diabetes	0.098 ***	0.030	0.071	0.053
L1*Cancer	0.056	0.064	0.293 ***	0.127
L1*Chronic lung diseases	0.078 ***	0.021	0.081 **	0.035
L1*Liver diseases	0.049 *	0.030	−0.036	0.050
L1*Heart diseases	0.074 ***	0.020	−0.009	0.036
L1*Kidney diseases	0.089 ***	0.026	0.115 ***	0.044
L1*Stomach or other digestive diseases	0.055 ***	0.014	0.020	0.023
L1*Arthritis or rheumatism	0.060 ***	0.012	0.016	0.020
L2*Hypertension			0.103 ***	0.025
L2*Diabetes			0.013	0.055
L2*Cancer			−0.037	0.108
L2*Chronic lung diseases			−0.018	0.037
L2*Liver diseases			0.111 **	0.055
L2*Heart diseases			0.020	0.038
L2*Kidney diseases			0.014	0.047
L2*Stomach or other digestive diseases			0.035	0.024
L2*Arthritis or rheumatism			0.036 *	0.021

Note: *** indicates *p* value < 0.01, ** indicates *p* value < 0.05, * indicates *p* value < 0.1; (1) and (2) indicate lag 1 and lag 2, respectively. “dy/dx” are the marginal effect coefficients of the probit regression on incidence of medical expenses.

**Table 6:** Heckman regression coefficients: Regression on total medical expenses.

	(1)		(2)	
	Coefficient	Std. Err.	Coefficient	Std. Err.
Lagging item of medical expenses				
L1 of medical exp. (ln)	0.225 ***	0.018	0.165 ***	0.022
L2 of medical exp. (ln)			0.113 ***	0.021
Predisposing factors				
Age (ref. 45–55)				
55–65	0.023	0.101	0 100	0.179
65–75	−0.002	0.113	0.229	0.187
≥75	0.088	0.160	0.447 *	0249
Gender (ref. male)	0.328 ***	0.084	0.297 **	0.132
Education (ref. less than lower secondary)				
Upper secondary, vocational training	0.089	0.131	−0.043	0.198
Tertiary	0.637	0.622	0.904	1.289
Marriage (ref. not)	0.258 **	0.123	0.252	0.189
Employ (ref. not)	−0.045	0.148	−0.164	0.208
Residence (ref. rural)	0.221 *	0.124	0.155	0.206
Enabling factors				
Number of outpatient visits (ref. ≤24)				
24–36	0.331 ***	0.097	0.203	0.152
≥36	0.827 ***	0.097	0.684 ***	0.150
Number of hospitalizations (ref. ≤1)	1.067 ***	0.142	0.911 ***	0.224
Income (ref. ≤CNY 8000)				
CNY 8000–15,600	0.042	0.157	0.009	0.254
CNY 15,600–30,000	0.284 *	0.166	0.391 *	0.227
≥CNY 30,000	0.307 *	0.167	0.330	0.239
Health insurance (ref. no)	2.734 ***	0.294	2.012 ***	0.344
Type of outpatient medical facilities (ref. general, specialized, Chinese medicine hospital)				
Community healthcare center, township hospital, village clinic	−1.167 ***	0.088	−1.151 ***	0.144
Multi-type outpatient facility visits (ref. no)	0.050	0.126	0.317	0.194
Need factors				
Hypertension	0.383 ***	0.103	0.315 **	0.154
Diabetes	0.408 ***	0.137	0.328 *	0.173
Cancer	0.088	0.320	0.535	0.489
Chronic lung diseases	0.220 **	0.107	−0.003	0.151
Liver diseases	0.013	0.153	−0.091	0.208
Heart diseases	0.346 ***	0.108	0.308 **	0.150
Kidney diseases	0.204	0.134	0.024	0.181
Stomach or other digestive diseases	0.057	0.095	0.029	0.144
Arthritis or rheumatism	0.197 **	0.095	0.222	0.146
Comorbidity (ref. ≤1)	0.274 **	0.131	0.654 ***	0.197
Self-reported health status (ref. very good)				
Good	1.334 ***	0.259	1.821 ***	0.418
Fair	1.149 ***	0.202	1.784 ***	0.353
Poor	1.388 ***	0.209	1.811 ***	0.368

Note: *** indicates *p* value < 0.01, ** indicates *p* value < 0.05, * indicates *p* value < 0.1; (1) and (2) indicate lag 1 and lag 2, respectively.

## Data Availability

The datasets used in the current study are not publicly available due to the confidential policy but are available from the corresponding author on reasonable request.

## Abbreviations

HCUs, high-cost users; CHARLS, the China Health and Retirement Longitudinal Study; PPS, probability proportionate to size; PSUs, primary sampling units. GDP, gross domestic product; GIS, geographic information system.
